# Supplementary data for the mechanism for cleavage of three typical glucosidic bonds induced by hydroxyl free radical

**DOI:** 10.1016/j.dib.2017.09.069

**Published:** 2017-10-02

**Authors:** Yujie Dai, Chunfu Shao, Yingai Piao, Huiqian Hu, Kui Lu, Tongcun Zhang, Xiuli Zhang, Shiru Jia, Min Wang, Shuli Man

**Affiliations:** a Key Laboratory of Industrial Fermentation Microbiology (Tianjin University of Science & Technology), Ministry of Education, College of Biotechnology, Tianjin University of Science and Technology, TEDA, No. 29 of 13th Street, Tianjin 300457, PR China; b Institute of Biology and Medicine, Wuhan University of Science and Technology, Huangjiahu Campus, Wuhan 430000, PR China; c Department of Biochemistry, University of Missouri, Columbia, MO 65211, USA

## Abstract

The data presented in this article are related to the research article entitled “The mechanism for cleavage of three typical glucosidic bonds induced by hydroxyl free radical” (Dai et al., 2017) [1]. This article includes the structures of three kinds of disaccharides such as maltose, fructose and cellobiose, the diagrammatic sketch of the hydrogen abstraction reaction of the disaccharides by hydroxyl radical, the structure of the transition states for pyran ring opening of moiety A and cleavage of α(1→2) glycosidic bond starting from the hydrogen abstraction of C6–H in moiety A of sucrose, the transition state structure for cleavage of α(1→2) glycosidic bond starting from the hydrogen abstraction of C1′-H in moiety B of sucrose, the transition state structure, sketch for the reaction process and relative energy change of the reaction pathway for direct cleavage of α(1→4) glycosidic bond starting from hydrogen abstraction of C6′–H of moiety B of maltose.

**Specifications Table**

**Table:** 

Subject area	*Chemistry*
More specific subject area	*Carbohydrate*
Type of data	*Graph, Figure, Text file*
How data was acquired	*By ChemBioDraw Ultra 12.0, Gaussian09 and Discovery Studio 2.5*
Data format	*Raw, Analysed*
Experimental factors	*Some transition state structures come from computation of Gaussian 09*
Data source location	*Tianjin, China.*
Data accessibility	*The data are available with this article*

**Value of the data**

● To facilitate the reader's understanding of this study.
● Extend readers' knowledge about the free radical reaction of carbohydrates.
● To lay a foundation for further study on the mechanism of polysaccharide degradation.

## 1 Data

**Fig. 1:**
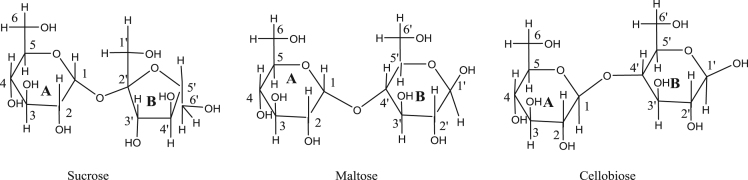
Structures of three kinds of disaccharides.

**Fig. 2:**
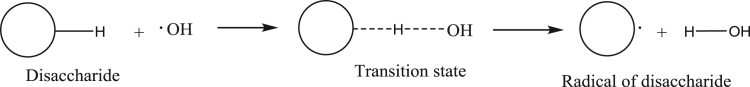
Hydrogen abstraction process of disaccharides by •OH.

Eight figures related to the research article entitled “The mechanism for cleavage of three typical glucosidic bonds induced by hydroxyl free radical” (Dai et al., 2017) [1] are included. The structures of three kinds of disaccharides such as maltose, fructose and cellobiose in Fig. 1, the hydrogen abstraction process of disaccharides by •OH in Fig. 2 and the direct cleavage of α(1→4) glycosidic bond from hydrogen abstraction of C6–H of moiety B of maltose in Fig. 5 were all sketched using ChemBioDraw Ultra 12.0. The relative energy change of the reaction pathway starting from hydrogen abstraction of C6–H in moiety B of maltose in Fig. 8 was generated by Origin 7.5. The 3D structures of transition states were generated by using BIOVIA Discovery Studio Visualizer 2016 [2] based on the TS optimization of the corresponding transition states with Gaussian 09 [3] at B3LYP/6–31+G(d,p) level [4], [5].

## 2 Experimental design, materials and methods

Data provided in this article are based on computation performed applying Gaussian 09 at B3LYP/6–31+G(d,p) level and are treated using ChemBioDraw Ultra 12.0, Origin 7.5 or BIOVIA Discovery Studio Visualizer 2016.

The chemical structures and the scheme of reaction pathways are shown below.

**Fig. 3:**
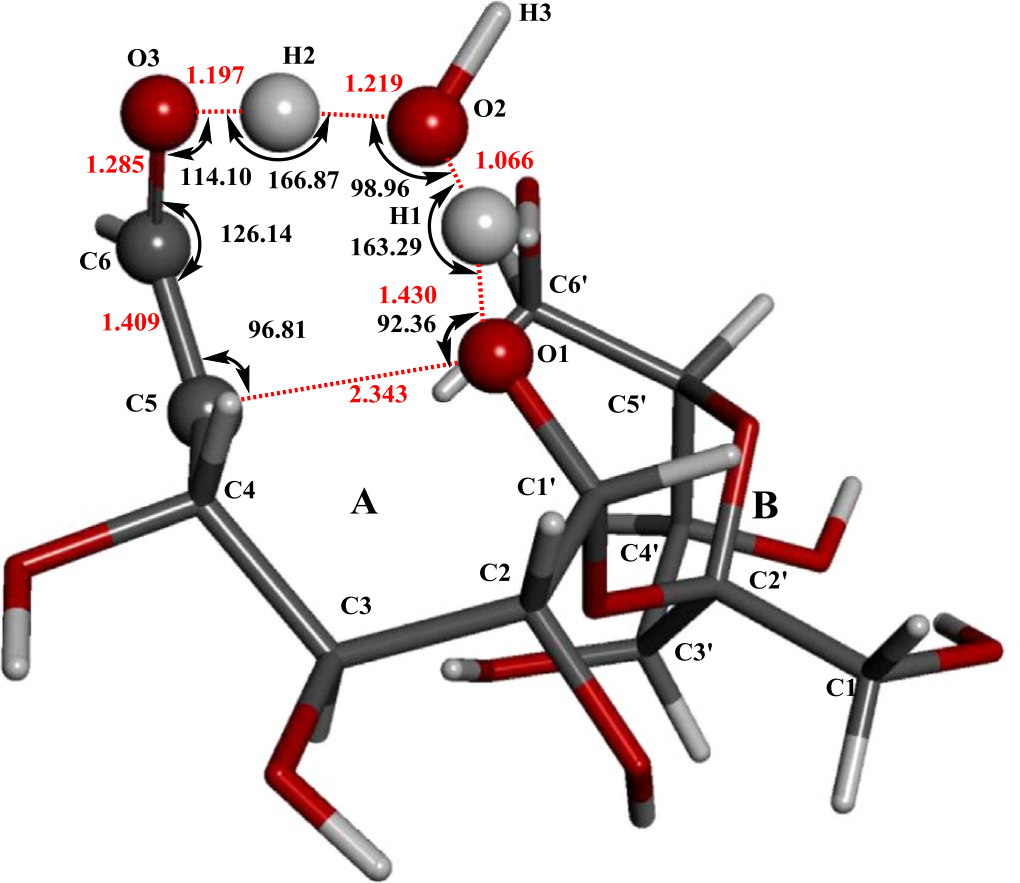
The structure of the transition state TS(II)_SuA6_ [The atoms shown in ball are that on the heptatomic ring of transition state. Distances (red), Å; Angles (black); H, white; O, red; C, grey].

**Fig. 4:**
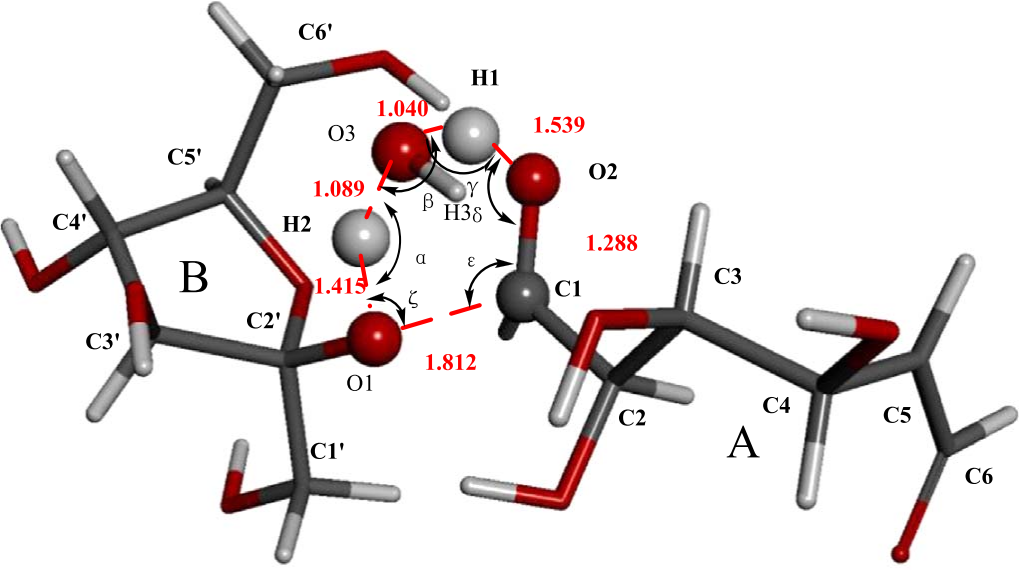
The structure of the transition state TS(III)_SuA6_ (The atoms shown in ball are that on the hexatomic ring of transition state. Distances, Å; Angles; H, white; O, red; C, grey; α,151.53;β,94.17;γ,149.50;δ,105.57;ε,107.36;ζ,96.79).

**Fig. 5:**
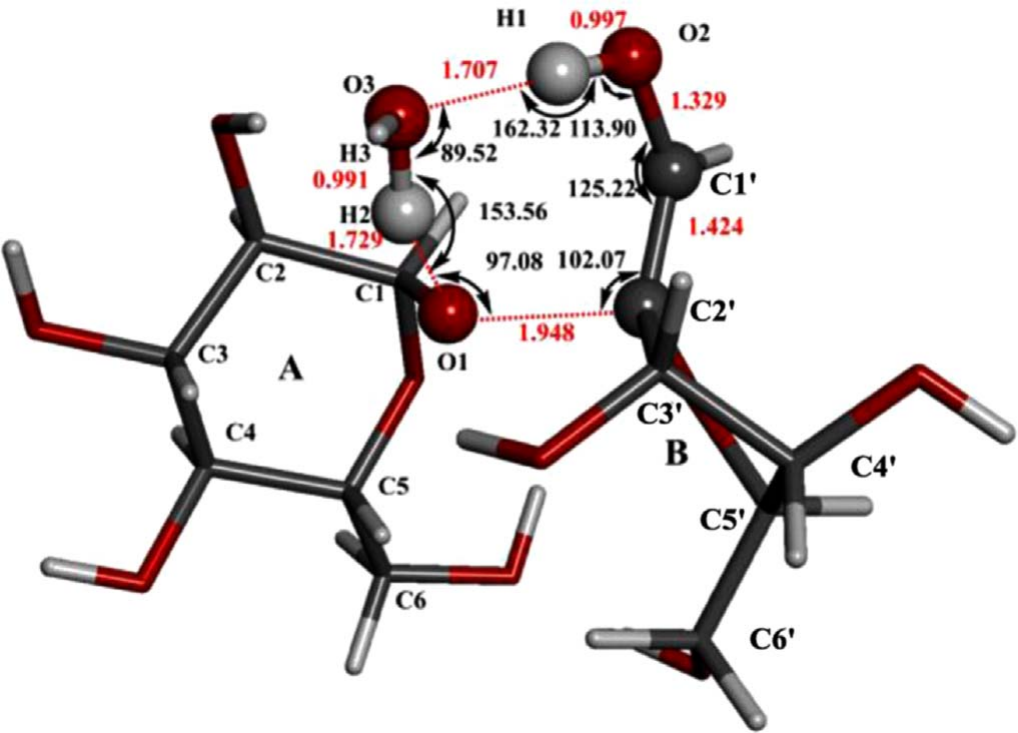
The structure of the transition state TS(II)_SuB1_ [The atoms in ball are on the heptatomic ring of transition state. Distances (red), Å; Angles (black); H, white; O, red; C, grey].

**Fig. 6:**
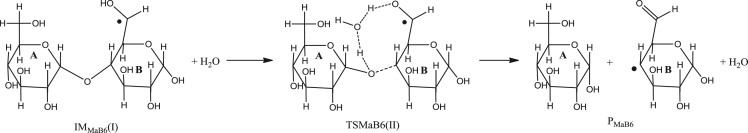
Direct cleavage of α(1→4) glycosidic bond from hydrogen abstraction of C6′–H of moiety B of maltose.

**Fig. 7:**
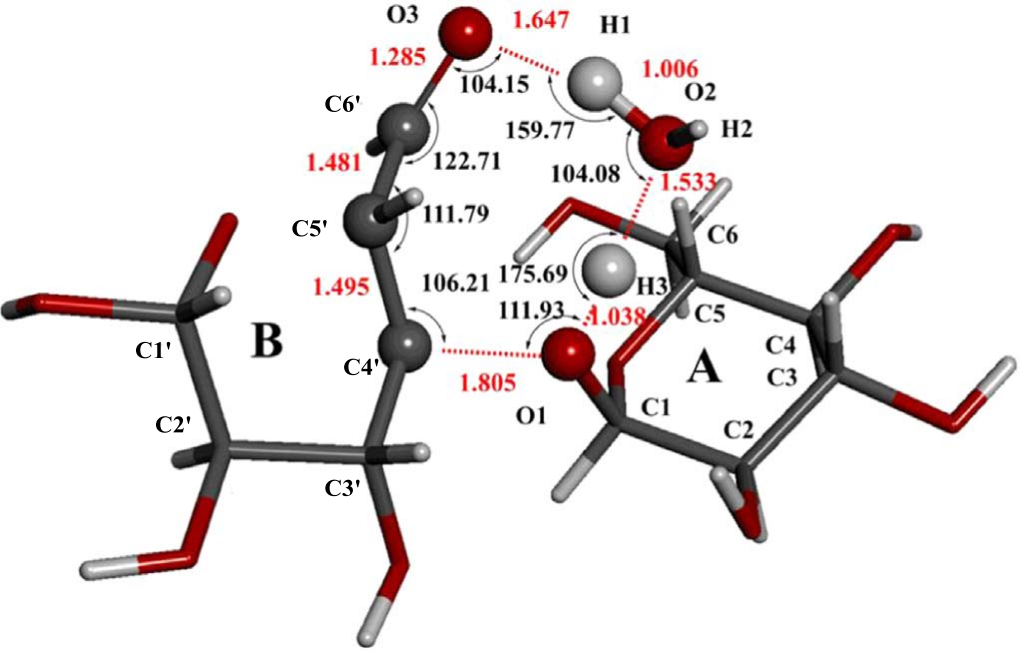
The structure of the transition state TS(II)_MaB6_ [The atoms in ball are on the heptatomic ring of transition state. Distances (red), Å; Angles (black); H, white; O, red; C, grey].

**Fig. 8:**
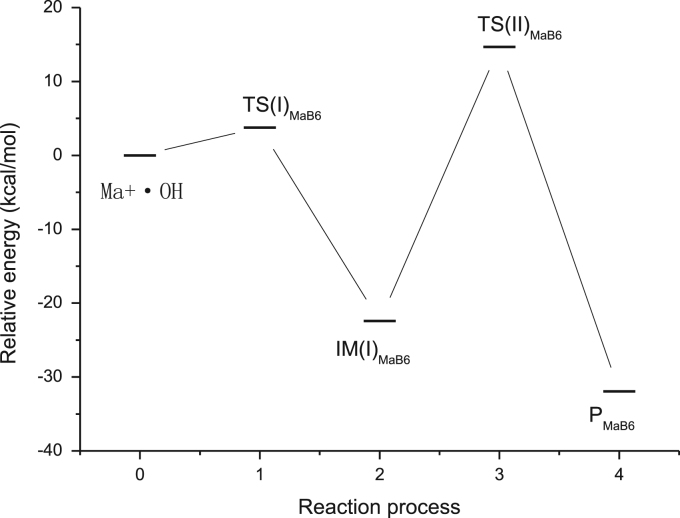
The relative energy change of the reaction pathway starting from hydrogen abstraction of C6′–H in moiety B of maltose by hydroxyl radical.

Fig. 1 shows the structures of sucrose, maltosse and cellobiose. The general hydrogen abstraction process of disaccharides by •OH is shown in Fig. 2. The structure of the transition state TS(II)_SuA6_, TS(III)_SuA6_, TS(II)_SuB1_ and TS(II)_MaB6_ were illustrated in Fig. 3, Fig. 4, Fig. 5, Fig. 7 respectively. The process of direct cleavage of α(1→4) glycosidic bond from hydrogen abstraction of C6′–H of moiety B of maltose is displayed in Fig. 6 and the plot of the relative energy change of the reaction pathway starting from hydrogen abstraction of C6′–H in moiety B of maltose by hydroxyl radical is shown in Fig. 8.
