# Supplementary material for: Supplementary data for the mechanism for cleavage of three typical glucosidic bonds induced by hydroxyl free radical
Source: Data Brief. 2017 Oct 2;15:414–8. doi: 10.1016/j.dib.2017.09.069 (PMC5712059; doi:10.1016/j.dib.2017.09.069)
Supplement: Supplementary file 1 — Transparency document [file mmc1.zip › CONFLICT OF INTEREST.pdf]

AUTHOR DECLARATION ABOUT CONFLICT OF INTEREST

We wish to draw the attention of the Editor to the following facts which may be considered as potential conflicts of interest and to significant financial contributions to this work. We wish to confirm that there are no known conflicts of interest associated with this manuscript entitled "**Supplementary data for the mechanism for cleavage of three typical glucosidic bonds induced by hydroxyl free radical**" for publication in the journal " **Data in Brief**" and there has been no significant financial support for this work that could have influenced its outcome.

We confirm that the manuscript has been read and approved by all named authors and that there are no other persons who satisfied the criteria for authorship but are not listed. We further confirm that the order of authors listed in the manuscript has been approved by all of us.

We confirm that we have given due consideration to the protection of intellectual property associated with this work and that there are no impediments to publication, including the timing of publication, with respect to intellectual property. In so doing we confirm that we have followed the regulations of our institutions concerning intellectual property.

We understand that the Corresponding Author is the sole contact for the Editorial process (including Editorial Manager and direct communications with the office). He is responsible for communicating with the other authors about progress, submissions of revisions and final approval of proofs. We confirm that we have provided a current, correct email address which is accessible by the Corresponding Author and which has been configured to accept email from yjdai@126.com (Yujie Dai).

Signed by all authors as follows:

Yujie Dai	Yujie Dai	Date:	2017. 9.12
Chunfu Shao	Chunfu Shao	Date:	2017.9.12
Yingai Piao	Yingai Piao	Date:	2017.9.12
Huiqian Hu	Huiqian Hu	Date:	2017.9.12
Kui Lu	Kui Lu	Date:	2017.9.12
Tongcun Zhang	Tongcun Zhang	Date:	2017 9.12
Xiuli Zhang	Xiuli Zhang	Date:	2017.9.12
Shiru Jia	Shiru Jia	Date:	2017. 9.12
Min Wang	Min Wang	Date:	2017. 9.12
Shuli Man	Shuli Man	Date:	2017.9.12
